# Assisted chanting and Buddhist end-of-life care in contemporary China

**DOI:** 10.1017/S1478951526103034

**Published:** 2026-07-13

**Authors:** Sophia Lynn Li, Jun Jing, Jun Zhang, Arthur Kleinman

**Affiliations:** 1 Harvard Medical School, Boston, MA, USA; 2 Department of Sociology, Tsinghua University, Beijing, China; 3 Department of Anthropology, Harvard University, Boston, MA, USA

**Keywords:** Palliative care, end-of-life care, spiritual care, Buddhism, medical anthropology, assisted chanting, China

## Abstract

**Objectives:**

The intersection of medicine and Buddhist spiritual care in institutional end-of-life settings remains underexplored, particularly regarding post-death rituals. This qualitative study examines how Buddhist assisted chanting (zhunian, 助念) – a 24-hour continuous recitation of Amitabha Buddha’s name – functions as a model of holistic end-of-life care that addresses the spiritual, emotional, psychological, and relational dimensions of dying.

**Methods:**

Semi-structured, in-person interviews were conducted with 34 participants (18 volunteers, 8 patients, and 8 family caregivers) at the Lotus Assisted Chanting Association housed within Cihai Hospital in Shenzhen, China, between June 22 and July 5, 2025. Interviews were conducted in Mandarin, transcribed, and anonymized. Coding was performed in NVivo N15 using an iterative thematic framework developed by open-coding an initial subset of transcripts.

**Results:**

Three care frameworks emerged: (1) *spiritual care*, including adherence to end-of-life Buddhist practices, acceptance of death, belief in rebirth, and release of worldly attachments; (2) *emotional and psychological care*, encompassing companionship, peace and tranquility, and financial burden alleviation; and (3) *reverse care*, in which dying patients exercised personal agency and tended to those around them by resisting over-medicalization, expressing concern for caregivers, and transforming caregiving into an opportunity to practice filial piety and strengthen faith.

**Significance of results:**

These findings characterize Buddhist assisted chanting as a reciprocal model of end-of-life care that extends care beyond the moment of death. Dying patients act as moral agents who tend to those around them, challenging the unidirectional caregiver–recipient model that underlies most palliative care research. These contributions show how culturally and spiritually informed palliative care prepares families for loss and shapes how patients find meaning at the end of life. More broadly, this study illustrates how Buddhist ritual frameworks can render dying as a humanistic and mindful process.

## Introduction

Death is a universal human experience (Aries 2013). Religious belief and spiritual practice help guide individuals during the end of life, providing them comfort and direction (Pentaris and Tripathi 2020). Across cultures, religious practice offers frameworks for understanding death, caring for the dying, and supporting families in mourning. Holistic palliative care encompasses not only physical, emotional, and psychological aspects but also the spiritual dimension of care – one deeply tied to the quality and meaning of people’s lives (Bacoanu et al. 2024).

China is home to a rapidly aging population. In 2022, more than 280 million people were aged 60 or older (Huld 2023). The World Health Organization predicts that this group will increase to more than one-fourth of the population by 2040, making end-of-life care an increasingly large focus for the country (Mo 2024; WHO n.d.). Buddhism is also a dominant religion, with estimates indicating that one-third of Chinese adults engage in Buddhist spiritual practices (Pew Research Center 2023).

Despite Buddhist religious prominence, current scholarship primarily focuses on biomedical models of palliative care in urban hospitals. For many patients, however, faith practices are integral to their end-of-life experience, and there is a paucity of scholarship examining how Buddhist spiritual frameworks shape caregiving, communal rituals, and end of life.

Pure Land Buddhism, one of the most widely practiced forms of Buddhism in China today (Deng 2017), centers its faith practice on attaining rebirth in the Western Pure Land of Bliss, a realm free from suffering (Zhou 2017). Early Buddhist texts, such as the Samyukta Āgama, describe how Amitabha comforted the sick and dying, teaching them how to attain rebirth and overcome death. Central to this practice is nianfo, or the chanting of Amitabha’s name. By chanting, practitioners help the dying cultivate a state of peace and mindfulness, let go of worldly attachments, and focus their energy on rebirth (Yu 2019).

These teachings were systematized by Master Yin Guan, the 13th patriarch of the Pure Land School of Chinese Buddhism, in his treatise Three Essentials at Life’s End (Guang 1936). He encouraged the establishment of spiritual aid associations, which have inspired modern-day zhuniantuan, or “assisted chanting,” associations. In these associations, Buddhist volunteers accompany the dying by chanting Amitabha’s name, providing spiritual guidance and easing their transition at the time of death. Yin Guan outlined 3 major principles: (1) provide comfort and strengthen the dying’s desire for rebirth in the Pure Land; (2) chant Amitabha’s name and encourage familial participation; and (3) avoid disturbing the deceased’s body and showing extreme emotion, as it may draw the deceased’s attention away from rebirth (Ye 2016; Chen 2021).

Assisted chanting aims to support the dying’s spiritual transition by fostering a community of care among fellow practitioners and by involving family members in the recitation (Chen 2018). It is carried out in temples, private households, hospices, and hospitals (Jing and Gao 2018). The ritual usually lasts for 24 continuous hours after medically-defined death, although it can range from 8 hours to over a full day, depending on physical signs such as the softness of the body and body temperature, which indicate a peaceful or successful passing (Ming 2014).

**Figure 1.**
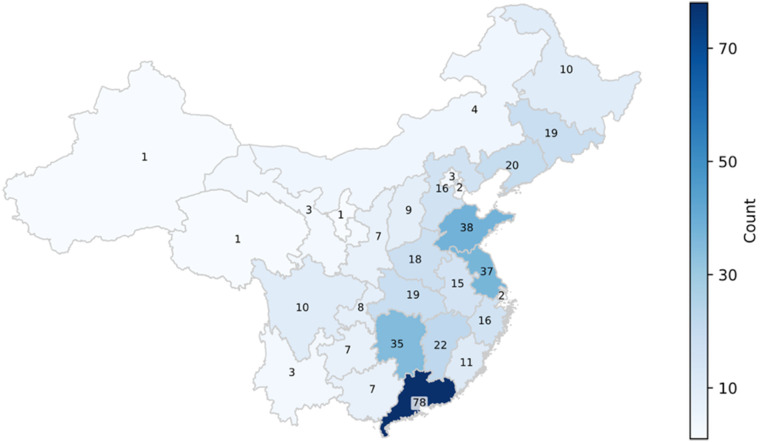
Distribution of assisted chanting associations across China’s provinces (Bie 2025).

Currently, there are an estimated 422 registered Buddhist assisted chanting associations nationwide (Figure 1). This study investigates Buddhist end-of-life care as practiced by the Lotus Assisted Chanting Association, part of the Lotus Life Care Volunteer Association (深圳市莲花生命关怀志愿者协会), a volunteer-based organization based at Cihai Hospital in Shenzhen, Guangdong. Cihai is a private institution founded in 2009 and houses the largest end-of-life care unit in its province. The Lotus Assisted Chanting Association comprises approximately 40 rotating Buddhist volunteers who provide end-of-life care by visiting Buddhist patients during their hospital stay and performing assisted chanting immediately post-mortem.

This study examines how assisted chanting serves as a form of care encompassing spiritual, emotional, psychological, and relational dimensions. Through qualitative fieldwork at Cihai Hospital – including interviews with patients, their families, and volunteers – this research explores how Buddhist end-of-life care is conducted in palliative care contexts. We addressed the following research questions: How do Buddhist patients and families make sense of death and dying? What forms of relational, spiritual, and emotional care do assisted chanting volunteers provide? What spiritual, psychological, and emotional transformations emerge from this shared care experience? By examining how modern Buddhist end-of-life care is performed, this study offers insight into how religious frameworks shape the experience of dying in contemporary China.

## Methods

For this qualitative study, a total of 34 participants (18 volunteers, 8 patients, and 8 family caregivers) were interviewed (Table 1). All interviews were conducted in-person at the Lotus Assisted Chanting Buddhist Care Facility at Cihai Hospital between June 22 and July 5, 2025.

**Table 1.** Characteristics of patients and respective family members interviewed Table 1 long description.

**Patient and family caregiver characteristics**				
Patient pseudonym	Gender	Age	Pseudonym (family caregiver)	Relationship (family caregiver)
Patient 1	M	67	F1	Son
			F2	Daughter
			F3	Daughter-in-law
Patient 2	F	68	F4	Son
Patient 3	M	80	F5	Aunt
			F6	Grandson
Patient 4	M	53	F7	Wife
			F8	Sister-in-law
Patient 5	F	47	–	–
Patient 6	F	68	–	–
Patient 7	F	82	–	–
Patient 8	M	56	–	–

– No family members of this patient were interviewed.

Patients or their family members signed a consent form to register for assisted chanting services. Once a hospital physician issued a death certificate, the patient’s body was transferred from the ward to the rebirth hall on their hospital bed, where the 24-hour assisted chanting practice began.

Semi-structured interviews focused on 4 core themes: (1) Daily life and sources of meaning and comfort (“Can you describe your daily routine and what brings it meaning?”); (2) Personal spiritual background (“What does your Buddhist practice look like?”); (3) Views on rebirth (“How do you understand death?”); and (4) Experiences of Buddhist ritual care (“How have you participated the Buddhist end-of-life care practices?”). Interview questions were posed to 3 participant groups (patients, family members, and volunteers), and interviews allowed for open-ended responses and follow-up prompts.

Participant recruitment for the study occurred on-site through direct fieldwork. Patients were interviewed during their hospital stay. While some passed away during or after the research period, not all did. Family members were interviewed either before or after a patient’s death.

Interviews were conducted in Mandarin, transcribed, and anonymized. Interviews were coded using NVivo 15. Audio recordings were deleted after transcription. A subset of 6 transcripts (2 volunteers, 2 patients, and 2 family members) was initially open-coded to develop a thematic framework. The coding scheme was refined in consultation with Z.J. and J.J. and then applied to the full dataset. Manual identification of themes and subthemes was conducted by S.L.L. and reviewed with the research team to ensure analytic consistency and rigor.

This study was conducted in accordance with the principles of the Declaration of Helsinki. Participants provided either written or oral consent following institutional ethical guidelines. All interviews were anonymized prior to analysis.

## Results

Qualitative analysis identified three care frameworks: (1) Spiritual care, encompassing Buddhist rituals and teachings related to death and rebirth; (2) Emotional and Psychological care, involving accompaniment and peace; and (3) Reverse care, focusing on patients’ impact on family members and volunteers (Table 2).

**Table 2.** Extracted and coded themes from interviews with patients, family members, and volunteers Table 2 long description.

**Coded themes**	
Themes	Subthemes
Spiritual care	Adhering to end-of-life Buddhist practices
	Accepting the inevitability of death
	Confirming belief in rebirth
	Letting go of worldly concerns
Emotional and psychological care	Providing companionship to families and the dying
	Encouraging peace and tranquility
	Alleviating financial burdens
Reverse care	Resisting over-medicalization
	Care for others
	Volunteer reflections

### Theme 1: Spiritual care

#### Adhering to end-of-life Buddhist practices

Volunteers at the Lotus Assisted Chanting Association support patients’ transitions to the Western Pure Land of Bliss through a wide range of spiritual practices. These include assisted chanting, striking a guiding bell, and performing merit-dedication rituals. They also educate patients and families about Buddhist views on death and the proper conditions for rebirth.

Within the first 24 hours, there are three key things. First is not moving the body. Once a person passes, we cannot move them, nor can we cry or wail. And we can’t change their clothes. We can’t move them. These three things are crucial. If our patients call, we immediately rush to the hospital room to begin the final recitation. Our role is to begin the final recitation and start our shifts. **Volunteer 9**

When a person dies, their soul starts wandering. Our final recitation serves to gather the soul, so they can hear every word we speak, every Amitabha we chant, every sound of the bell we strike.… According to Buddhism, we provide them with the Amitabha recitation to lessen their suffering, including helping calm their spirit and ease their pain. **Volunteer 5**

Volunteers also provide families with guidance on preparing the deceased for rebirth. This involves explaining the spiritual criteria and giving them an opportunity to participate in a structured farewell.

We tell family caregivers that the deceased elder must meet three conditions. First is believing that Amitabha can save them from suffering. Then, being willing to go to the Western Pure Land and enjoying its fulfillment—gold-paved ground and wishes granted at the thought, wanting clothes and receiving clothes, wanting food and receiving food. Lastly, they must let go. During the communication stage with the family, we give them an introduction and encourage them to use their own words in a ‘Verbal Farewell’ segment. This ‘Verbal Farewell,’ includes gratitude, acknowledging the deceased, and then, if there are any regrets—for example, being unable to always be by their side—apologizing to the deceased. Saying ‘please forgive me, thank you, we will never ever forget you.’ Through our communication process, helping them assist the deceased to let go of what they were attached to, everything will be taken care of. **Volunteer 1**

#### Accepting the inevitability of death

Part of Pure Land Buddhist practice is accepting that death is a natural and inevitable transition rather than something to be feared.

I don’t think death is a bad thing. It is a very natural process. It can happen in old age and while someone is very young. Death is a natural part of life. Before, my understanding was solely from books, but now I can see it for myself. It helps me understand death on a deeper level. **Volunteer 5**

Some people are healthy their whole lives, full of happiness. Some people live ordinary lives, others face hardships, but every person will reach their life’s end. Everyone has a moment when they close their eyes for the last time. **Volunteer 10**

#### Confirming belief in rebirth

A central tenet of Pure Land Buddhism is the cycle of reincarnation and rebirth. Death is the beginning of new life, just as life is the end of death.

Actually, death isn’t scary. After learning Buddhism, we know where we will go. **Volunteer 11**

While participating in assisted chanting, family caregivers expressed similar beliefs:

My understanding of end-of-life care is that this is but the physical world. People will go to the lotus afterlife. The body is empty, breath is empty—don’t be confused. The body will decay, but the soul is eternal. **Wife of Patient 4 (F7)**

Patients themselves spoke about their desire for rebirth:

I don’t want it. Even if I could live on and have a lot of money, I wouldn’t want it. I want to be reborn in the Western Pure Land. **Patient 8 (M, 56)**

#### Letting go of worldly concerns

To successfully reincarnate in the Western Pure Land, the patient must remove worldly attachments through the process of “fangxia,” literally to set down or let go. This principle requires not only detachment from material belongings and past accomplishments but also from relationships. Lingering attachments are believed to hinder rebirth.

Our volunteer chanting group is there to strengthen the deceased’s faith, to help them be reborn without obstacles, to let go of everything tied to their body and mind, including any attachments. Including their attachments to the elders, their material goods.… Our chanting group helps them let go of everything in this life so they can have peace of body and mind. **Volunteer 5**

If a patient’s body remains stiff after 24 hours, volunteers will extend the assisted chanting ritual until the body becomes soft.

If we need to add additional assisted chanting time, we feel a bit uneasy. Why is that? Because sometimes the elder could not let go of certain things. Each thing they cling to means we must add time. Only after it’s fully resolved can they pass. Seeing that they require more time, makes us feel bad. It just feels—did we not do a good enough job? Why can’t they let go? We feel worried. **Volunteer 9**

Letting go is not only for the deceased. Family caregivers are encouraged to confront their own emotional attachments as well.

I can’t live forever with sorrow and self-blame. Don’t cling too tightly to anything. Don’t have fixations. Letting go of them changes your mindset. Through this ritual, my heart feels like it has been cleansed. I can let go a bit more. **Daughter of Patient 1 (F2)**

Common worries patients harbored were related to pain, financial burdens, family affairs, and past relationships. They described how Buddhist teachings helped them relinquish their anxieties and move toward a more peaceful state of mind.

Just comparatively, I’m not afraid—not terrified of death, terrified of pain, scared of pain. There’s a lot of overthinking, thinking about how much money I’m spending, thinking about memories from my childhood—all types of anxieties. After learning Buddhism, these things started to change. It helped me find inner peace.… I invite the Buddha to take me away. To let me leave earlier to go to the Western Pure Land. **Patient 5 (F, 47)**

After I started believing in Buddhism, I’ve freed myself from worries and have a stronger sense of non-self and non-ego.… Cihai Hospital’s Buddhist faith model is very good. The body can’t carry anything with it. Your mind is the most important. Before, I would really miss my daughter and husband. Now I don’t miss them anymore. **Patient 6 (F, 68)**

### Theme 2: Emotional and psychological care

#### Providing companionship to families and the dying

Participants spoke about how accompaniment is integral to caring for patients at the end of life.

I’ve realized that in end-of-life care, the meaning of accompaniment isn’t necessarily *what* we do, but in the simple act of *being there*. As life comes ashore, it’s about providing a quiet harbour, accepting vulnerability, silence, and the natural course of things. To use our presence to bear witness to someone else’s needs and to help another lonely soul by lighting a small flame to illuminate the world. **Volunteer 4**

When a patient is admitted to a hospital, we must first care for them, not wait until they pass to start doing things. That’s ineffective. You must care for people beforehand. You must visit these elderly Buddhas’ hospital rooms often.… You have to chat with them, show concern, and visit them often, you know? Foundational care is like this. If you have the chance, the time, you can visit. Honestly, you can bring them downstairs for some sunshine. Right? This is *life* care, to care for others. Then they will trust you. **Volunteer 7**.

Family caregivers also reflected on their experiences:

When people reach the end of life, accompaniment is the best form of care. What matters most in accompaniment is helping the dying go without fear, with peace of mind. **Sister-in-law of Patient 4 (F8)**

In other hospitals, there were only cold, mechanical processes—it felt icy. The rituals here are comforting. **Daughter of Patient 1 (F2)**

#### Encouraging peace and tranquility

Volunteers describe how chanting rituals help create a soothing environment for both the dying and their families.

End-of-life care can help the dying person leave with peace and calm while remaining dignified. It can also help family caregivers find comfort, peace, and avoid regret. It lessens their fear of death and strengthens family harmony and unity—so the living and the dead are both at peace. **Volunteer 1**

Family caregivers echoed the sense of peace they felt:

I think having assisted chanting rituals brings family caregivers comfort. It calms them. **Aunt of Patient 3 (F5)**

Patients also described their impressions of the volunteer community:

Yesterday, when I came down, I saw everyone. I saw you all. Everyone’s smiles were very nice. And you were all so compassionate and kind. It just gave me a feeling that coming here, I could feel safe. **Patient 5 (F, 47)**

#### Alleviating financial burdens

End-of-life care is often associated with significant financial strain for families, including the cost of treatment, funeral rites, and burial arrangements. The Lotus Assisted Chanting Association rituals are completely free. During the chanting period, families are not charged for care, food, lodging, or even burial clothes. The absence of financial pressure not only makes care accessible but also brings emotional and psychological reassurance, allowing families to focus on their loved ones.

A lot of people in big cities live in apartment complexes. If you have a relative pass away at home, some people worry that neighbors will be afraid. If they pass in the hospital, ordinary wards won’t take patients like that. They can only live in the ICU. However, the costs are very high. There is a need for hospitals that offer end-of-life care. **Daughter of Patient 1 (F2)**

Patients also cited the financial feasibility of the arrangements:

It is affordable. I can stay. We can afford the costs. **Patient 6 [F, 68]**

### Theme 3: Reverse care

Just as Buddhist views on life, death, and rebirth are cyclical, the care context in assisted chanting associations is interconnected and reciprocal. Buddhist scholar Da Zhao and Jing Jun establish a framework for thinking not only about how volunteers and families support the dying but also about how the terminally ill tend to those around them in what they term “reverse care” (Jing and Song 2024).

#### Over-medicalization resistance

In contrast to a biomedical model that emphasizes prolonging life through aggressive interventions, volunteers described an approach grounded in Buddhist teachings. Rather than resisting death at all costs, they emphasize comfort and dignity, not wanting to overburden their families after death. They viewed it as a reallocation of hospital resources or care that would better serve others. A volunteer described the Buddhist philosophy behind their views on treatment plans:

When we speak about Western medicine, when someone reaches their end of life, just like a teacher said before, don’t deliberately try to prolong their life. That’s a type of life with no quality.… If it’s time to go, it’s best to go without burdens. **Volunteer 2**

Patients echoed these sentiments, demonstrating care and concern for those around them:

I want to pass soon. I don’t want any of my loved ones suffering with me. When one person is sick, those caring for them are also suffering. **Patient 5 (F, 47)**

Whether I can extend my life is unimportant now. Here, we give and are surrounded by care, by love. **Patient (F, 68)**

#### Care for others

Patients often expressed care for the well-being of the volunteers by asking them to sit, reminding them to walk slowly, or praying for their safety. One elder, though bedridden, often asked the Buddha to protect those around them:

Buddha protects me, Buddha protects everyone. **Patient 7 (F, 82)**

Patients also described extending verbal comfort to their families and reassuring them:

I’m at peace. My sister was crying, and I said, ‘Here, there might be a miracle. If there’s a miracle, that’s good. If there isn’t, I’ll go to the Western Pure Land.’ I said, ‘There, I can still come back. When that time comes, I can come and help you all—help you all be better.’ So, my sister’s mood has improved a lot these past two days. **Patient 5 (F, 47)**

Caring for a dying parent was also seen as a chance for children to practice filial devotion and accrue karmic merit. Their very act of being cared for was understood not as a burden but as a spiritual opportunity, as one volunteer explained:

By being here, you are helping your children accumulate merit... Your son is here to repay your great kindness. You gave him life, so he will accompany you. Being by your side is a way to repay your kindness. Your hardship gives them a chance to express their gratitude. Stay here and let them be filial. **Volunteer 12**

#### Volunteer reflections

Volunteers found that being close to death transformed their personal spiritual outlooks. Patients gave them a chance to contribute to a mutually caring environment, something seen as a gift rather than an obligation. One volunteer described the reciprocal nature of rebirth in the Western Pure Land:

I’ve helped many Buddhas attain rebirth. Honestly, I’m very happy. I really am very happy. Why? Later, after we’ve helped send off these elderly people, they’ll become Buddhas and receive us, you know? **Volunteer 7**

Working with patients has also shaped volunteers’ own faiths and understanding of palliative care:

Experiencing another person’s death helps us transcend life and death. Encountering another person’s death will help us stop fearing death and build an unwavering faith for rebirth in the Western Pure Land. **Volunteer 1**

‘End-of-life care’: these four words are often seen separately. ‘End-of-life’ describes the patients; ‘care’ describes us. But I don’t think it’s just that. I once accompanied an elderly woman… Faith gave her strength. Her eyes had light inside them. We illuminated each other. End-of-life care is about illuminating each other. It flows. **Volunteer 10**

## Discussion

Western models of end-of-life care typically end at the moment of death, whereas Buddhist care extends beyond, supporting patients and their families before and especially after passing (Fang and Qi 2018). This study of the Lotus Assisted Chanting Association at Cihai Hospital illustrates how Buddhist frameworks and medical settings intersect to produce a unique and holistic model of end-of-life care. The 24-hour assisted chanting ritual not only provides spiritual guidance – emphasizing Buddhist values such as letting go of worldly attachments and strengthening faith in rebirth (Tian 2018) – but also addresses emotional and psychological needs, through accompaniment and the removal of financial stressors associated with end-of-life proceedings. Families also found comfort, companionship, and space to process the loss of a loved one.

Patients cultivated a peaceful mindset around death, sustained by their faith in rebirth and by practicing compassion. The Buddhist emphasis on reverse care enabled them to remain active participants in their final days, providing verbal and spiritual support to family members and volunteers. Their very presence provided opportunities for care to be given and received.

Perspectives from non-Buddhist patients or staff were not included, nor were those of physicians or nurses, which may have provided valuable additional insights. Despite these limitations, the study demonstrates how Buddhist moral and ritual frameworks shape end-of-life experiences and caregiving practices.

These findings also have implications for palliative care beyond China. With a growing literature on Buddhist chaplaincy contexts, for example, in the United States, the principles in this study can inform broader hospice and palliative care practices (Wang 2017). In contrast to conventional medical routines, in which bodies are sent to the morgue shortly after death, Buddhist practices re-center dying as a humanistic and mindful process – one that takes up time, energy, and space.

Religion generally assists mourning by providing a template and form of practice for the deeply personal experience of grief (Watson and Rawski 1990). Anthropologists, psychologists, historians, and religious scholars have repeatedly shown that mourning practices not only strengthen personal and family memories of those who have passed, thereby assisting the grieving process, but also enable communities and societies to remember (Oyebode and Owens 2013). Because patients described in this paper had been in end-of-life care in a hospital, families had time for anticipatory grieving (Raphael 1994). Remembering those who have passed enables communities and societies to go on and to revivify (Connerton 2014). The model of care observed at Cihai Hospital shows how Buddhist moral frameworks and ritual practices can reshape not only how death is approached but also how end-of-life care is given and received (Kleinman 2019).

## Table 1 Long description

The table lists eight patients by pseudonym with gender and age, and links each patient to any interviewed family caregivers by caregiver pseudonym and relationship. Patients include four men and four women, ranging in age from 47 to 82 years. Four patients have at least one interviewed caregiver: Patient 1 (male, 67) has three caregivers, a son, a daughter, and a daughter-in-law; Patient 2 (female, 68) has one caregiver, a son; Patient 3 (male, 80) has two caregivers, an aunt and a grandson; Patient 4 (male, 53) has two caregivers, a wife and a sister-in-law. The remaining four patients, Patients 5 through 8, have no interviewed family caregivers recorded. Caregiver relationships span immediate family and extended family, and the table reflects only those family members who were interviewed rather than all possible caregivers.

Navigate back to Table 1.

## Table 2 Long description

The table organizes coded interview themes into three main themes with associated sub-themes. Spiritual care includes adhering to end-of-life Buddhist practices, accepting the inevitability of death, confirming belief in rebirth, and letting go of worldly concerns. Emotional and psychological care includes providing companionship to families and the dying, encouraging peace and tranquility, and alleviating financial burdens. Reverse care includes resisting over-medicalization, care for others, and volunteer reflections. The content is categorical and descriptive, listing topics rather than counts or rankings, and does not indicate how often each theme occurred.

Navigate back to Table 2.
